# Development of Microsatellite Markers for the Nipa Palm Hispid Beetle, *Octodonta nipae* (Maulik)

**DOI:** 10.1155/2018/9139306

**Published:** 2018-06-07

**Authors:** Zhiming Chen, Jun Chen, Xiang Zhang, Youming Hou, Guihua Wang

**Affiliations:** ^1^State Key Laboratory of Ecological Pest Control of Fujian-Taiwan Crops, Fujian Agriculture and Forestry University, Fuzhou 350002, China; ^2^Fujian Provincial Key Laboratory of Insect Ecology, College of Plant Protection, Fujian Agriculture and Forestry University, Fuzhou 350002, China; ^3^Fuzhou Entry-Exit Inspection and Quarantine Bureau of P.R.C, Fuzhou 350002, China

## Abstract

The nipa palm hispid beetle, *Octodonta nipae* (Maulik) (Coleoptera: Chrysomelidae), is an important invasive pest on palm plants in southern China. Based on existing transcriptome data, polymorphism simple sequence repeat (SSR) loci were identified. In total, 1274 SSR loci were identified from 49919 unigenes. The majority of them contained mononucleotide, dinucleotide, and trinucleotide motifs (43.56%, 26.14%, and 28.18%), in which A/T (41.21%) and AT/TA (15.86%) were the most abundant motifs. 104 pairs of the SSR primers produced amplification bands of expected sizes in *O. nipae*, 80 pairs of SSR primers were tested randomly for polymorphism, 9 loci of them were validated to be polymorphic markers, and the number of alleles ranged from 2 to 3, with an average of 2.56 per locus. The population of Zhangzhou and Fuzhou was analyzed by the 9 loci (On1–On9). These SSR transcriptome data can provide invaluable resource for SSR development, population genetics research, invasion and expansion mechanism, paternity testing, and other research on *O. nipae* and its related species.

## 1. Introduction

The nipa palm hispid beetle, *Octodonta nipae* (Maulik) (Coleoptera: Chrysomelidae), which was originated in Malaysia, is an alien pest on palm plants in southern China [1]. The beetles attack young leaves of various palm plants, which poses a great threat to palm plants industry, economic palm planting, city landscaping, and ecological safety [2].

Much efforts have been devoted to research on *O. nipae*, including external morphology, physiology characteristic, biological habits, raising management, disease and pest control technique [3–6], multiple mating [7], transcriptome immune analysis [8], and rapid identification of species based on cytochrome c oxidase subunit I (COI) and internal transcribed spacer (ITS) [9]. Genetic markers are largely lacking for *O. nipae*; in recent years, microsatellites, also called simple sequence repeats (SSRs), have been widely used in studies of intra- or interspecies variation and genetic structure in biological populations because of their high levels of polymorphism, codominant Mendelian inheritance, and ease of detection by polymerase chain reaction [10]. Recently, less microsatellite DNA has been reported in *O. nipae* and its closely related species, excepting eight microsatellite DNA loci described for use in *Brontispa longissima* [11]. However, the lack of microsatellite markers developed specifically for *O. nipae* has caused a bottleneck in the study of population genetics in *O. nipae*.

In this study, a series of microsatellite markers for *O. nipae* were developed. The utility of the newly developed nine markers were tested in two Fujian populations (Zhangzhou and Fuzhou). These polymorphism microsatellite markers would be powerful tools for pests' population genetic and dispersal studies in the future. It is the first time that microsatellite markers have been utilized in *O. nipae*.

## 2. Materials and Methods

### 2.1. Insects Sampling and DNA Extraction

*O. nipae* collected from canary date palm *Phoenix canariensis* in Zhangzhou (North latitude 24.3074, East longitude 117.6833) and Fuzhou (North latitude 25.7186, East longitude 119.3612), Fujian Province, were bred in laboratory as previously described [12]. The total DNA of adults from two populations (Zhangzhou and Fuzhou) was extracted individually using the DNA extraction kit (SK8252, Sangon Biotech Co., Ltd., Shanghai, China) according to the manufacturer's instructions.

### 2.2. Microsatellite Development

Based on the existing transcriptome data of *O. nipae* [4], TRINITY software was used to screen all perfect microsatellites [13]. The evaluation criterion used for mono-, di-, tri-, tetra-, penta-, and hexanucleotide repeats was the default setting in TRINITY, with a minimum repeats of 12, 6, 5, 5, 4, and 4, respectively. After searching the *O. nipae* genome, primers were designed in Primer Premier3.0 (http://www.sourceforge.net/projects/primer3/files/primer3/1.1.4/primer3-1.1.4-WINXP.zip/download) for possible high polymorphism, and less than 100 reached the scoring size standards. Microsatellites with mononucleotide repeats were removed, when primers were designed. All primers were designed according to the following parameters: product size ranged from 100 to 400 bp, primer size ranged from 18 to 28 bp, and the optimum size was about 23 bp. All primers were synthesized by the Beijing Genomics Institute (BGI, Shenzhen, China). The primer pairs were evaluated by Oligo 7 and confirmed at each optimum annealing temperature.

To examine those primer amplification effects, we amplified single locus by PCR. The 25 *μ*L reaction volume contains 12.5 *μ*L 2 × Taq PCR MasterMix (TIANGEN), 9.5 *μ*L ddH_2_O, 1 *μ*L forward primer (10 pmol), 1 *μ*L reverse primer (10 pmol), and 1 *μ*L DNA template. The thermal profile used an initial denaturation step of 94°C for 3 min followed by 35 cycles of denaturation at 94°C for 30 s, annealing at optimum temperature for 30 s, and extension at 72°C for 40 s. A 5 min final extension at 72°C was added at the end of cycle. PCR products were checked on 2% AGE (agarose gel electrophoresis).

Fluorescent genotyping method was used to select appropriate loci for further polymorphism examination. We amplified single locus by PCR. The PCR amplification program was the same as above, apart from the DNAs of 10 individuals used as the PCR templates, and the forward primer of each pair was labeled with a fluorescent dye FAM or HEX or ROX. PCR products were analyzed using ABI 3130 sequencer (Applied Biosystems) according to the manufacturer's instructions. Allele sizes were determined using GENEMAPPER version 4.0 (Applied Biosystems), LIZ-500 as size standard.

The number of alleles per locus (NA), expected heterozygosity (*H*_*e*_), observed heterozygosity (*H*_*o*_), and polymorphic information content (PIC) were assessed using Microsatellite Tools for Excel [14]. Hardy–Weinberg equilibrium (HWE) and genotypic linkage disequilibrium between pairs of microsatellites were calculated with GENEPOP V3.4 [15]. This method precludes the false positive in multiple tests. When the loci deviated from HWE, tests were performed using GENEPOP V3.4 to find out whether deviations were the result of a deficit or an excess of heterozygotes.

## 3. Results

### 3.1. Characterization of SSR Distribution

1274 SSR loci were identified from the 49919 unigenes. These SSRs have 555 mono-, 333 di-, 359 tri-, 17 tetra-, 6 penta-, and 4 hexanucleotide repeats, which corresponded to 43.56%, 26.14%, 28.18%, 1.33%, 0.47%, and 0.31% of total SSRs, respectively. Frequency of SSRs based on the number of repeat units in *Octodonta nipae* transcriptome is provided in Table 1.

The 10 most frequent motif types in transcriptome were A/T (41.21%), AT/TA (15.86%), AAG/CTT (7.61%), AAT/ATT (7.22%), AC/GT (5.49%), AG/CT (4.79%), AAC/GTT (3.45%), ACC/GGT (3.14%), ATC/ATG (2.43%), and C/G (2.35%). All the possible cases were included, while calculating the frequency of each motif type. In a repeat sequence, for example, AC contained AC and CA, and AAC have AAC, ACA, and CAA. The frequency and number of repeats of all microsatellite motif types are given in Table 2.

### 3.2. Microsatellite Characteristics

By analyzing the 1274 SSR loci by the software Primer Premier 5, we found 157 loci were suitable for designing 788 pairs of primers. According to the evaluation of Oligo 7.0, the primers of 124 loci were up to standard, namely, 40 for dinucleotide repeats, 80 for trinucleotide repeats, and 4 for tetranucleotide repeats, respectively. In total, 124 pairs of primers were designed and synthesized, one optimized pair of primers for one locus.

Out of the 124 primer pairs, 104 primer pairs amplified the expected products. The 9 loci that appeared to be polymorphic and easy to amplify were chosen to genotype 24 individuals from 2 different populations (12 individuals from Zhangzhou population and 12 individuals from Fuzhou population). The number of alleles per locus ranged from 2 to 3, with an average of 2.56. The primer sequences and other summarized data for each locus are given in Table 3.

In Zhangzhou population, the expected and observed heterozygosity ranged from 0.083 (On1) to 0.681 (On2) and from 0.083 (On1) to 0.667(On1), respectively. Out of the 9 loci, three (On1, On3, and On8) were significantly deviated from HWE. Further analyses revealed that On1 was caused by excess of homozygotes, and On3 and On8 were due to excess of heterozygotes. The PIC (polymorphic information content) of alleles per locus ranged from 0.077 to 0.584, with an average of 0.372 (Table 3).

In Fuzhou population, the expected and observed heterozygosity ranged from 0 (On4 and On6) to 0.591 (On2) and from 0 (On4 and On6) to 0.583 (On18), respectively. Three of the 9 loci (On2, On4, and On6) were significantly deviated from HWE. Further analyses revealed that those 3 loci were caused by excess of homozygotes. The PIC of alleles per locus ranged from 0 to 0.501, with an average of 0.256 (Table 3).

## 4. Discussion

In this study, we found that the SSR loci of *O. nipae* had some difference with those of other insects, which can be observed from Table 4, and the ratio of mononucleotide repeat was mostly in the transcriptome data of the four known insects, ranging from 37.59% to 89.44% with significant difference. In general, except for mononucleotide repeat, trinucleotide repeat was dominant. Because the codon was constituted of 3 nucleotides, the loss and increase of one trinucleotide repeat would not result in frameshift mutations while the loss and increase of mononucleotide, dinucleotide, and tetranucleotide repeat would result in severe frameshift mutations, which further affected the expression and regulation of genes [16]. The ratio of dinucleotide repeat in *O. nipae* was about 26.14% similar to the ratio of trinucleotide repeat (28.18%) which was different with the situation in other insects. This kind of situation may be caused by the different screening criteria of SSR and transcriptome cover.

A/T repeat unit was abundant in the SSR repeat unit of *O. nipae*: A/T, AT/TA, and AAG/GTT/AAT/ATT were the dominant types of repeat unit of mononucleotide, dinucleotide, and trinucleotide, respectively. It can be seen from this that the SSR had a preference of the sequence enriched of A or T; therefore, the frequency of SSR sequence enriched of G or C was relatively low, and thus, our results are in line with the previously published research results of transcriptome [17]. As the SSR of transcriptome was located in the transcribed region, the sequence of SSR would affect the translation to perform different function. The study of the SSR enriched of A or T would deepen the understanding of the function of SSR. Moreover, the polymorphism of SSR in animals and plants was positively correlated with the length of SSR [18], generally the polymorphism of low-grade units was higher than that of high-grade units [19], the polymorphism of dinucleotide repeat and trinucleotide repeat in repetition of 8 bases or more may be higher, according to the abovementioned criteria, the number of SSR loci meeting with the conditions of dinucleotide repeat and trinucleotide repeat was 103 and 11, which possessed the ratio of 30.9% and 3% in dinucleotide repeat and trinucleotide repeat, respectively. According to this, we speculated that the polymorphism detection efficiency ofdinucleotide repeat of *O. nipae* would be higher than that of trinucleotide. Our experimental results verify the speculation that 7 out of 9 polymorphic sites are dinucleotide repeat. It means that, in order to improve the development efficiency of the SSR polymorphic site in transcriptome data, we selected proper site by detecting the abundance and distribution of SSR.

Up to February 2017, the transcriptome data of more than 400 kinds of insect were uploaded to NCBI which provides lots of precious resources for SSR development. Although the development of SSR based on transcriptome sequencing was used more and more widely, it still has many shortcomings [20–22]: (1) The polymorphism of the SSR obtained from the transcriptome was lower than that of the SSR obtained from the genome, even some transcriptome SSRs have no polymorphism, because the polymorphism of SSR is related to the times of repetition of core sequence, but the times of repetition of transcriptome SSR is not high. (2) The SSR obtained from transcriptome is cDNA without introns, and it is different from the corresponding genome SSR; thus, the length of PCR products may be longer than expected; the PCR amplification may be inefficient or the PCR amplification may fail. (3) Defect still exists in high-throughput sequencing assembly which can lead to many SSRs with more repetitions fracturing or missing in the assembly process, leading to the transcriptome data incomplete and less reliable.

Setting more effective criteria to screen SSR based on the characters of SSR was suggested, for instance, assessment of flank sequence length and similarity could be added into and sites with more repetitions could be chosen in the process of site selection [23]. The development efficiency could be improved greatly by means of strict biological information method. Besides, it is very meaningful to explore the SSR function of molecular markers, through gene location and sequence alignment conduct function annotation to get a deeper understanding of the biological meaning of these genes [24]. As part of the transcriptome SSR can be used in the same genus and even within the same family to conduct cross-species amplification, those chosen sites can be used to construct an evolutionary tree model to analyze evolution and classification and other issues.

The 9 loci that appeared to be polymorphic and easy to amplify were chosen to genotype 24 individuals from two different populations (Fuzhou and Zhangzhou populations), and their PIC had large differences in those two populations. Some loci were discovered to be significantly deviated from HWE (*P* < 0.05), which may be due to population bottleneck effect or null alleles or small sample size, considering the fact that the species invaded Fujian Province several years ago. The relatively short time for the species invasion into Fujian may result in the low allele diversity in this study. Although the polymorphisms of 9 loci were not very high, they should be powerful tools for investigating the genetic diversity and the invasion route in *O. nipae*.

## Figures and Tables

**Table 1:** Frequency of SSRs based on the number of repeat units in *Octodonta nipae* transcriptome.

Repeat type	Number of repeats									Total	Percentage
	4	5	6	7	8	9	10	11	≥12		
Mononucleotide	—	—	—	—	—	—	—	—	555	555	43.56
Dinucleotide	—	—	159	71	43	18	20	22	—	333	26.14
Trinucleotide	—	255	78	15	11	—	—	—	—	359	28.18
Tetranucleotide	—	14	3	—	—	—	—	—	—	17	1.33
Pentanucleotide	6	—	—	—	—	—	—	—	—	6	0.47
Hexanucleotide	4	—	—	—	—	—	—	—	—	4	0.31
Total	10	269	240	86	54	18	20	22	555		
Percentage	0.78	21.11	18.84	6.75	4.24	1.41	1.57	1.73	43.56		

**Table 2:** Frequency of SSRs based on motif types in *Octodonta nipae* transcriptome.

SSR	Number of repeats									Total	Percentage
SSR motif type	4	5	6	7	8	9	10	11	≥12		
A/T	—	—	—	—	—	—	—	—	525	525	41.21
C/G	—	—	—	—	—	—	—	—	30	30	2.35
AC/GT	—	—	28	17	14	5	4	2		70	5.49
AG/CT	—	—	28	9	10	4	4	6		61	4.79
AT/TA	—	—	103	45	19	9	12	14		202	15.86
AAC/GTT	—	33	9	1	1	—	—	—		44	3.45
AAG/CTT	—	63	23	6	5	—	—	—		97	7.61
AAT/ATT	—	70	17	2	3	—	—	—		92	7.22
ACC/GGT	—	28	9	3	—	—	—	—		40	3.14
ACG/CGT	—	2	1	1	—	—	—	—		4	0.31
ACT/AGT	—	8	2	1	2	—	—	—		13	1.02
AGC/CTG	—	14	5	1	—	—	—	—		20	1.57
AGG/CCT	—	11	1	—	—	—	—	—		12	0.94
ATC/ATG	—	24	7	—	—	—	—	—		31	2.43
CCG/CGG	—	2	4	—	—	—	—	—		6	0.47
AAAC/GTTT	—	1	—	—	—	—	—	—		1	0.08
AAAG/CTTT	—	1	—	—	—	—	—	—		1	0.08
AAAT/ATTT	—	6	—	—	—	—	—	—		6	0.47
AACC/GGTT	—	3	—	—	—	—	—	—		3	0.24
AAGT/ACTT	—	1	1	—	—	—	—	—		2	0.16
AATC/ATTG	—	1	—	—	—	—	—	—		1	0.08
ACTG/AGTC	—	—	2	—	—	—	—	—		2	0.16
AGAT/ATCT	—	1	—	—	—	—	—	—		1	0.08
AAAAT/ATTTT	3	—	—	—	—	—	—	—		3	0.24
AAACT/AGTTT	1	—	—	—	—	—	—	—		1	0.08
AACCT/AGGTT	1	—	—	—	—	—	—	—		1	0.08
AATGT/ACATT	1	—	—	—	—	—	—	—		1	0.08
AAATTC/AATTTG	2	—	—	—	—	—	—	—		2	0.16
AATGGG/ATTCCC	1	—	—	—	—	—	—	—		1	0.08
AGCGGC/CCGCTG	1	—	—	—	—	—	—	—		1	0.08

**Table 3:** Characteristics of 9 microsatellite loci isolated from *Octodonta nipae*.

Locus	Primer sequences (5′-3′)	Repeat motif	*T*_A_ (°C)	Range (bp)	*N*_A_	*H*_*e*_		*H*_*o*_		PIC		HWE	
						ZZ	FZ	ZZ	FZ	ZZ	FZ	ZZ	FZ
On1	F: AACATAGAATCGACATCATGCGT R: GATTTACAGAACGGAGTTCATGC	(TA)8	47.6	96–98	2	0.083	0.344	0.083	0.250	0.077	0.275	—	0.404
On2	F: AGTTGCATAACTTCCGAAATGAA R: TCTAGGAATCAGCTCTGCCATTA	(GT)9	52.4	99–103	3	0.681	0.591	0.667	0.250	0.579	0.501	0.398	0.002
On3	F: GCTTTGCAACAACAAAGAAGAAT R: TCAAAAGAAAGGAAAAAGTTCCC	(CA)10	50.9	97–105	3	0.678	0.359	0.750	0.417	0.584	0.307	0.015	1.000
On4	F: AGACGTCAAGTTTTAACACGCAT R: TATAGACGGAGAACTGGCAACAT	(AT)6	53.7	113–117	2	0.159	0.000	0.167	0.000	0.141	0.001	1.000	—
On5	F: TGCTTAGATTTAGCACCAAGCTG R: TTAAATTCGAACCAAGCTGGTAG	(GT)8	53.1	131–137	3	0.562	0.522	0.583	0.333	0.432	0.375	1.000	0.282
On6	F: AGCTAATTGCGGCTGATTAGATA R: GCGGTAAATATGTCAAAAGCAAG	(AT)6	50.2	159–161	2	0.159	0.000	0.167	0.000	0.141	0.001	1.000	—
On7	F: ATGTGTACCCTGAAAGAGGGTTT R: GAAAAATTTGAGAAGCTTCGGTT	(CT)7	51.3	155–163	3	0.663	0.453	0.417	0.417	0.561	0.369	0.065	1.000
On8	F: AATGAAAAATTGACTTGGCAGAA R: CCGGCGTACATAACCTACTTTTT	(AAG)5	51	121–127	3	0.344	0.431	0.417	0.583	0.275	0.328	0.001	1.000
On9	F: AACTTAAACCAGAATCGGCTCTC R: ATGATGTCGGACGATTCTCTTC	(CAA)5	55	138–141	2	0.663	0.163	0.167	0.167	0.561	0.150	1.000	0.487

*T*_A_, primer annealing temperature; range, size range of alleles in base pairs; *N*_A_, number of alleles per locus; *H*_*e*_, expected heterozygosity; *H*_*o*_, observed heterozygosity; PIC, polymorphic information content; HWE, Hardy–Weinberg equilibrium.

**Table 4:** Frequency of SSRs based on the number of repeat units in four insects' transcriptome.

Insect	Percentage					
	Mononucleotide	Dinucleotide	Trinucleotide	Tetranucleotide	Pentanucleotide	Hexanucleotide
*Octodonta nipae*	43.56	26.14	28.18	1.33	0.47	0.31
*Phenacoccus solenopsis*	89.44	2.31	7.52	0.67	0.06	—
*Nilaparvata lugens*	37.59	17.28	37.38	2.09	0.52	0.21
*Tenebrio molitor*	44.44	11.21	41.15	1.68	0.96	0.56
